# Transcriptional and Metabolic Responses of Maize Shoots to Long-Term Potassium Deficiency

**DOI:** 10.3389/fpls.2022.922581

**Published:** 2022-06-23

**Authors:** Wangdan Xiong, Yujian Wang, Yongzhen Guo, Wei Tang, Yiran Zhao, Guofeng Yang, Yuhe Pei, Jingtang Chen, Xiyun Song, Juan Sun

**Affiliations:** ^1^Grassland Agri-husbandry Research Center, College of Grassland Science, Qingdao Agricultural University, Qingdao, China; ^2^College of Agriculture, Qingdao Agricultural University, Qingdao, China

**Keywords:** silage maize, potassium, biomass yield, metabolome, transcriptome

## Abstract

Potassium is important for plant growth and crop yield. However, the effects of potassium (K^+^) deficiency on silage maize biomass yield and how maize shoot feedback mechanisms of K^+^ deficiency regulate whole plant growth remains largely unknown. Here, the study aims to explore the maize growth, transcriptional and metabolic responses of shoots to long-term potassium deficiency. Under the K^+^ insufficiency condition, the biomass yield of silage maize decreased. The transcriptome data showed that there were 922 and 1,107 differential expression genes in DH605 and Z58, respectively. In the two varieties, 390 differently expressed overlapping genes were similarly regulated. These genes were considered the fundamental responses to K^+^ deficiency in maize shoots. Many stress-induced genes are involved in transport, primary and secondary metabolism, regulation, and other processes, which are involved in K^+^ acquisition and homeostasis. Metabolic profiles indicated that most amino acids, phenolic acids, organic acids, and alkaloids were accumulated in shoots under K^+^ deficiency conditions and part of the sugars and sugar alcohols also increased. It revealed that putrescine and putrescine derivatives were specifically accumulated under the K^+^ deficiency condition, which may play a role in the feedback regulation of shoot growth. These results confirmed the importance of K^+^ on silage maize production and provided a deeper insight into the responses to K^+^ deficiency in maize shoots.

## Introduction

Potassium (K^+^) is one of the essential macronutrients required for plant growth and development, such as photosynthesis, osmoregulation, enzyme activation, protein synthesis, and ion homeostasis (Kanai et al., 2011; Hafsi et al., 2014). A large area of cropland has low levels of K^+^, and crops cannot efficiently utilize the mineral elements in the soil (Perry et al., 1972; Hafsi et al., 2014). Adding K^+^ fertilizer can increase crop yield, but the intensified use of K^+^ fertilizer in agriculture gives rise to environmental pollution (Römheld and Kirkby, 2010). From the year 1961 to 2015, the estimated K^+^ utilization efficiency of cereal crops was only 19%, underscoring the need to conserve this non-renewable natural resource (Dhillon et al., 2019). Therefore, improving the K^+^ utilization efficiency of crop plants is crucial for optimizing fertilizer use, improving crop yield, and reducing environmental pollution. To optimize the K^+^ utilization of specific crop species, it is important to investigate the response and adaptations of crops to K^+^ deficiency and its underlying mechanisms.

The potassium is absorbed by plant roots and transported to the aboveground tissues, in which K^+^ transporters and channels play important roles in absorbing and transporting potassium (Jia et al., 2008). Under K^+^ deficiency conditions, plants change their root structure and modify their root hairs to absorb more nutrients (Jia et al., 2008). Recently, the transcriptome profiles of plant roots under K^+^ deficiency conditions have been analyzed in rice, wheat, soybean, cotton, and tomato (Ma et al., 2012; Ruan et al., 2015; Singh and Reddy, 2017; Zhao et al., 2018; Yang et al., 2021). Genes involved in metabolic pathways, such as carbohydrates, plant hormones, and kinases, play indispensable roles in maintaining plant growth under K^+^ deficiency conditions, and transcription factors (Hyun et al., 2014; Zhao et al., 2018; Yang et al., 2021). Moreover, the shoot can feedback on regulating the uptake of mineral nutrients (such as nitrogen and phosphate) from roots (Bari et al., 2006; Tabata et al., 2014). Regulators, such as high-affinity K^+^ transporter, were identified to be associated with shoot regulation of root K^+^ uptake in response to its deficiency in cotton (Wang Y. et al., 2019). Thus, it is important to analyze how the plant shoots adapt under K^+^ deficiency as well.

Maize (*Zea mays* L.) is an important multi-functional crop in food, animal feed, and energy production (Schnable, 2012). Besides, maize can be used as silage maize, which is an important feed for intense ruminant production, exhibiting high biomass production, and relatively low input demand (Baghdadi et al., 2018). The growth of maize relies heavily on the use of chemical fertilizers, besides potassium (Baghdadi et al., 2018). Potassium deficiency puts abiotic stress on crops, which restricts their yield (Zorb et al., 2014; Qin et al., 2019). Plants would respond to potassium deficiency at different levels (Hafsi et al., 2014). Low potassium levels would induce lateral root growth in maize, in which genes associated with nutrient utilization, hormones, and transcription factors are involved (Zhao et al., 2016; Ma et al., 2020). Although the root system underlies potassium absorption, it is unclear how low K^+^ concentration induces the expression of related genes and metabolites in maize shoots to maintain its shoot growth and promote root absorption to accommodate K^+^ deficiency.

In this study, the growth of silage maize was analyzed with K^+^ treatment in the hydroponic experiment. Furthermore, transcriptional and metabolic profiles of shoots were investigated for their effects on maize development under the K^+^ deficiency condition. The study was to determine the primary effects of K^+^ deficiency on the growth of silage maize and to explore the potential transcriptional and metabolic responses in shoots. This study helps to understand the physiological adaptation mechanisms of corn silage development under K^+^ deficiency conditions and provides a theoretical basis for improving the nutrient utilization efficiency of silage maize in breeding.

## Materials and Methods

### Plant Growth and Sample Collection for Hydroponic Experiment

For the hydroponic experiment, two varieties (DH605 and Z58) were used to investigate their responses to K^+^ deficiency. DH605, a common local maize variety grown in China, is used as a grain and forage maize. The inbred line Z58 has a high general combining ability and is suitable for breeding and preparing hybrids. Maize seeds were surface sterilized in 10% H_2_O_2_ for 20 min, rinsed, and then germinated in coarse quartz sand until two leaves were visible. The seedlings were grown in the chamber for 3 days (16 h light/8 h dark, 25°C, 60% relative humidity) and cultured with modified Hoagland solution (Zhao et al., 2012). The seedlings were transferred to a nutrient treatment solution containing either 0.1 mM KCl (K^+^ deficiency; LK) or 4 mM KCl (K^+^ sufficiency; CK) to assess their response. The solution was refreshed every 3 days and the treatment lasted for 12 days. There were three biological replicates. Plant shoots used for K^+^ concentration, metabolic and transcriptional analyses were immediately frozen in liquid nitrogen and then stored at −80°C until use.

### The Photosynthetic Rate in Leaves and Measurement of Soluble K^+^ Concentration

The photosynthetic rate of the uppermost newly and fully expanded leaves was measured with an LI-6800 portable photosynthesis system (LI-COR Inc., Lincoln, NE, USA) after 12 days of treatment. The photosynthetically active radiation was set to 800 μmol.m^−2^.s^−1^. The freeze-dried and powered shoot samples (0.2 g) were used to analyze the K^+^ concentration using a flame photometer (Model 410, Sherwood Scientific Ltd, Cambridge, UK) following the previously described H_2_SO_4_-H_2_O_2_ decoction method (Kanai et al., 2007).

### RNA Isolation, QPCR, and Transcriptome Analysis

Total RNA was isolated using Trizol reagent (Invitrogen, Carlsbad, CA, USA) and RNA quality and integrity were determined using Nanodrop 2000 (Thermo, Waltham, Massachusetts, USA). For RNA sequencing (RNA-seq) library synthesis, three biological replicates per treatment were sequenced with an Illumina HiSeq-PE150 instrument. Gene expression levels were normalized by calculating reads per kilo base of transcript per million fragments mapped (RPKM) using HISAT2 2.0.5 after mapping to the B73 reference genome sequences (Zm-B73-REFERENCE-NAM-5.0; https://ftp.ncbi.nlm.nih.gov/genomes/all/GCF/902/167/145/GCF_902167145.1_Zm-B73-REFERENCE-NAM-5.0/). The absolute value of log2 (fold change) ≥ 1 (treatment/control) (*p* ≤ 0.05) was defined as differentially expressed gene transcripts (DEGs). DEGs were also employed for Gene Ontology (Go) and Kyoto Encyclopedia of Genes and Genomes (KEGG) analysis.

### Metabolite Measurements

To analyze the metabolite under K^+^ deficiency, metabolite abundances were extracted and quantified at Wuhan MetWare Biotechnology Co., Ltd. (www.metware.cn) (Wang S. et al., 2019). The maize shoots were freeze-dried, powdered, and then extracted with 70% v/v aqueous methanol at 4°C. The extractions were centrifugated at 12,000 *g* for 10 min and then filtered (0.22 μm pore size). The obtained supernatants were analyzed using a UPLC-MS/MS system (UPLC, SHIMADZU Nexera X2; MS/MS, Applied Biosystems 4500 QTRAP). Principal component analysis (PCA) was used to analyze the differential accumulated metabolites (DAMs) between samples of K^+^ treatments. The relative importance of metabolites was checked with the parameter of variable importance in the project (VIP). Metabolites with VIP ≥ 1 and an absolute value of log_2_ (fold change) ≥ 1 (treatment/control) were considered as DAM.

### Data Analysis

Data on plant stem diameter, fresh weight, yield, K^+^ concentration, and photosynthetic rate were examined using SPSS software. Significance was defined as the probability level of the Student's *t*-test at *p* ≤ 0.05.

## Results

### Effect of K^+^ Deficiency on Maize Growth

**Figure 1:**
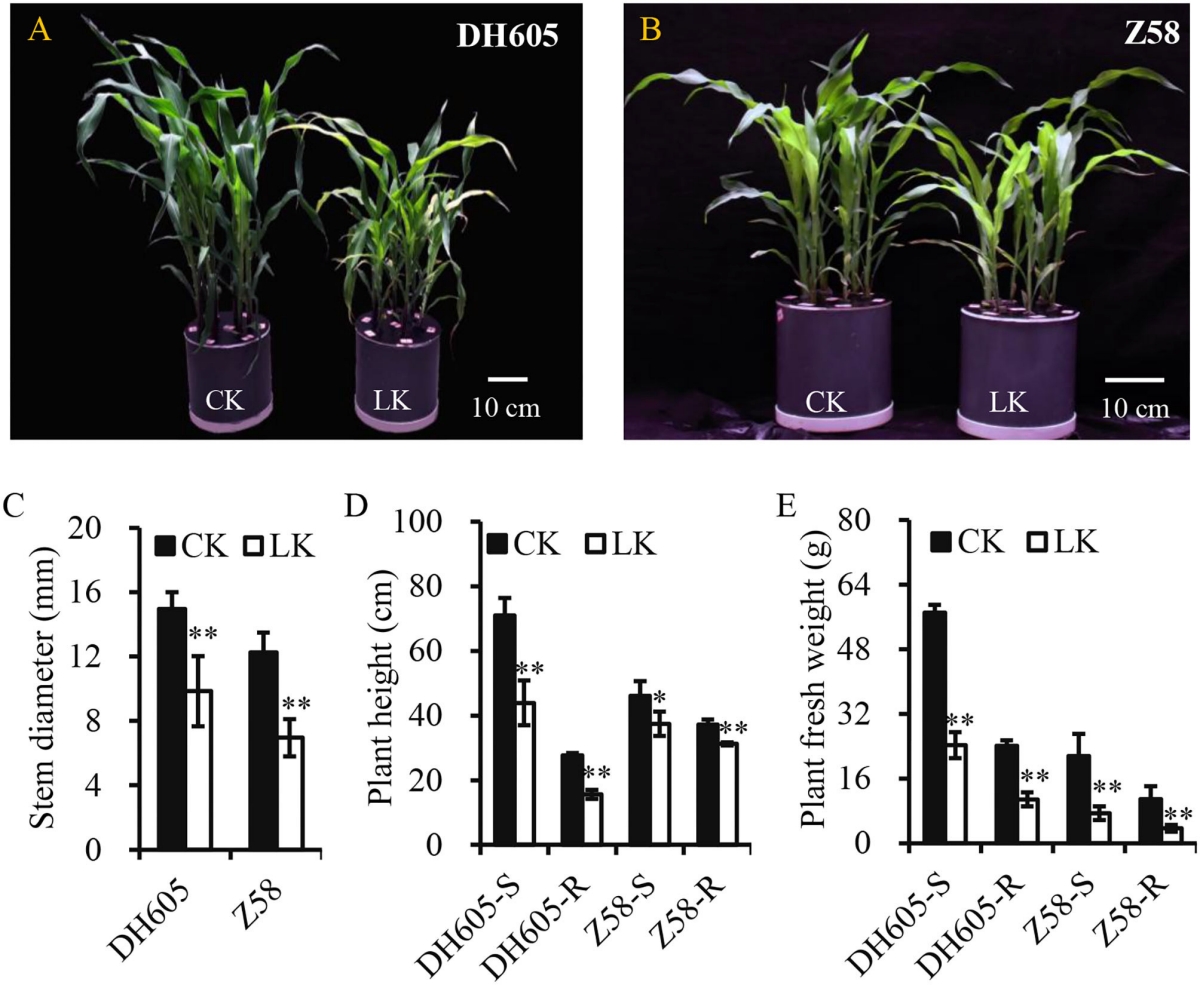
Effect of K^+^ deficiency on maize growth. **(A,B)** Showed phenotypes of maize DH605 and Z58 under K^+^ treatment for 12 days, respectively. **(C–E)** showed the variations of average stem diameter, plant height, and plant fresh weight under K^+^ deficiency condition for 12 days, respectively. Two maize varieties, DH605 and Z58, were analyzed to K^+^ deficiency, respectively. CK and LK represent K^+^ sufficiency (4 mM) and K^+^ deficiency (0.1 mM). Scale bars in **(A,B)** represent 10 cm. The experiments were repeated three times. ^*^ and ^**^ on histograms mean the significant difference at the *p*≤0.05 and *p*≤0.01 level, respectively. Bars mean SD.

**Figure 2:**
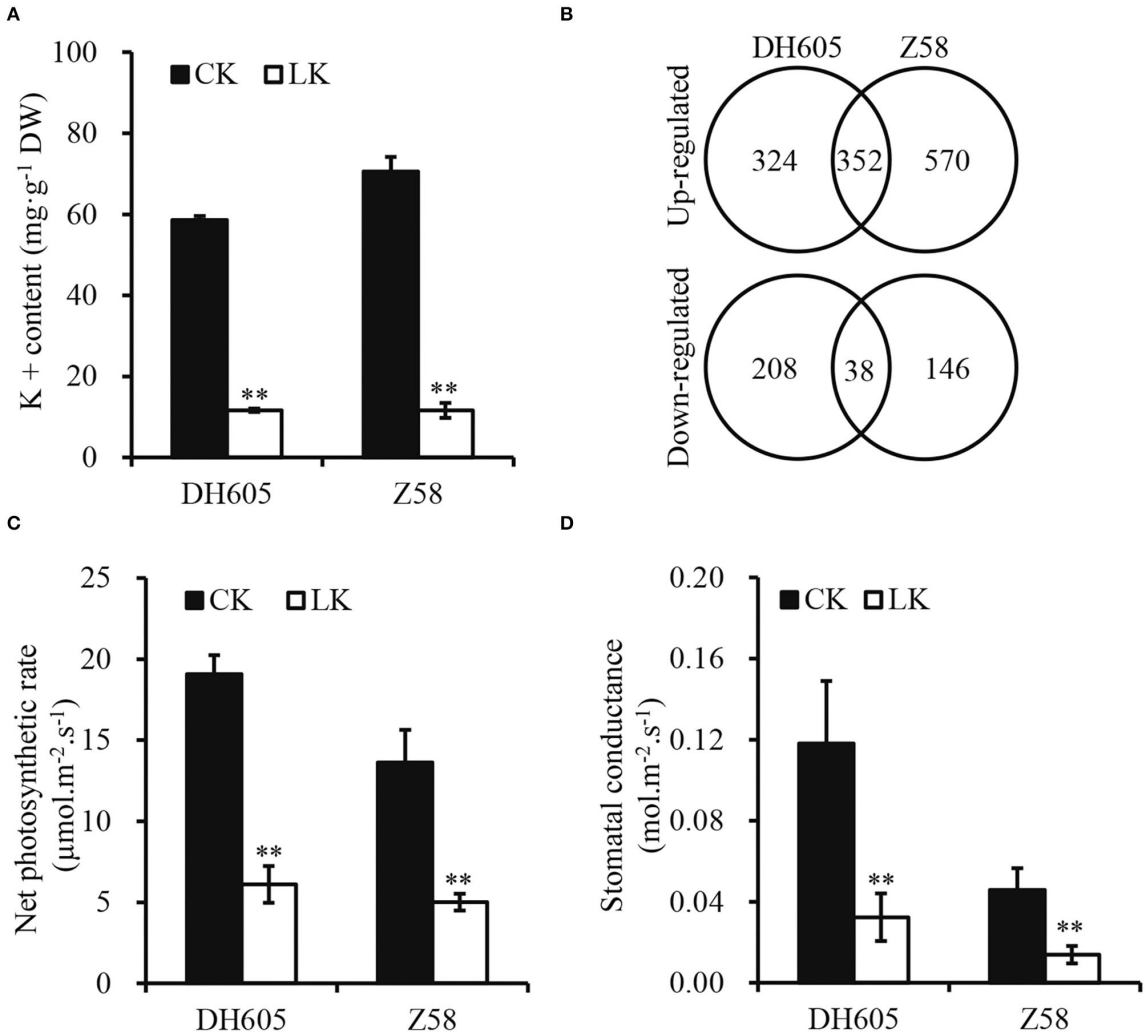
Shoot potassium content **(A)**, differential expression genes **(B)**, leaf net photosynthetic rate **(C)**, and stomatal conductance **(D)** of maize DH605 and Z58 under K^+^ deficiency treatment. CK and LK represent K^+^ sufficiency (4 mM) and K^+^ deficiency (0.1 mM), respectively. The experiments were repeated three times. ^**^ on histograms mean the significant difference at the *p* ≤ 0.05 and *p* ≤ 0.01 level, respectively. Bars mean SD.

To investigate the mechanism of K^+^ deficiency for maize growth, two varieties (DH605 and Z58) were used to investigate their responses to K^+^ deficiency (0.1 mM) in the hydroponic experiment. The results showed that K^+^ deficiency significantly decreased the shoot height and root length of both varieties and their stems were slender (Figures 1A–D). Moreover, the fresh weight of shoots significantly decreased by 57.5 and 65.5% in DH605 and Z58, respectively, under K^+^ deficiency conditions and the root fresh weight also showed a similar trend (Figure 1E). The K^+^ deficiency in the hydroponic solution resulted in a larger reduction in the K^+^ content of shoots, with the K^+^ concentration reduced by 80.16 and 83.54% in DH605 and Z58, respectively (Figure 2A). Under the K^+^ deficiency condition, maize's net photosynthetic rate displayed a dramatic reduction in both varieties compared to plants grown under the K^+^ sufficiency condition (Figure 2B).

### Global Transcriptional Changes in Response to K^+^ Deficiency

To obtain a global overview of the transcriptome relevant to K^+^ deficiency in maize, RNA-seq libraries from shoot samples of DH605 and Z58 were set up after 12 days of treatment under the K^+^ deficiency condition. After the removal of low-quality reads, an average of 4.5 × 10^7^ clean reads were obtained for each sample, and the total length of the clean reads reached above 6.2 × 10^9^ nt. For each sample, 97.5% of the clean reads were mapped to the maize reference transcriptome (Supplementary Table 1). A total of 35,733 genes were mapped to the genome. The DEGs were 922 in DH605, including 676 upregulated and 246 downregulated genes (Figure 2C, Supplementary Table 2a). In total, 1,106 DEGs were detected in Z58, including 922 upregulated and 184 downregulated genes (Figure 2C, Supplementary Table 2b). Moreover, 352 genes were upregulated in both DH605 and Z58, and the number of co-downregulated genes was only 38 (Figure 2C). Among the co-regulated DEGs, the expression of specific K^+^ transporters-related genes was induced in a K^+^ deficient environment (Table 1). The expression levels of two high-affinity K^+^ transporters (*HAK*) genes (*LOC100502520-HAK1* and *LOC100384472-HAK5*) were up-regulated under the K^+^ deficiency condition. Gene encoding inward potassium channels (*LOC100281406-AKT2*) was also upregulated to promote uptake of K^+^ both in maize shoots. But, *HAK10* and *HAK11* were downregulated under the K^+^ deficiency condition. Moreover, ten genes encoding ABC transporters were also upregulated for both two varieties, which are reported to be involved in the transport of mineral and organic ions, amino acids, oligosaccharides, lipids, and metal ions (Table 1; Xie et al., 2020).

**Table 1:** Genes encoding transporters showed differential expression in response to K^+^ deficiency (0.1 mM).

**Gene family**	**Seq ID**	**DH605**	**Z58**	**Gene description**
ABC transporter	*LOC100274125*	1.74	1.36	ABC transporter C family member 3
	*LOC100286314*	2.67	3.17	ABC transporter B family member 2-like
	*LOC100381832*	2.01	1.55	ABC transporter C family member 4
	*LOC100384044*	1.97	2.85	ABC transporter G family member 36
	*LOC100501068*	2.23	1.38	ABC transporter G family member 9-like
	*LOC103633239*	2.19	2.55	ABC transporter G family member 43
	*LOC103636202*	2.46	3.66	ABC transporter B family member 11
	*LOC103641937*	2.90	3.76	ABC transporter C family member 15
	*LOC103652531*	2.52	1.91	ABC transporter B family member 9
	*LOC103653413*	5.22	5.11	ABC transporter A family member 7
AKT	*LOC100281406*	2.36	1.92	Potassium channel AKT2
HAK/KT/KUP	*LOC100502520*	1.61	1.51	HAK1
	*LOC100384472*	4.38	4.97	HAK5
	*LOC100281081*	−1.82	−1.34	Potassium transporter HAK10
	*LOC103645946*	−1.72	−1.79	Potassium transporter HAK11

*The value indicates increase or decrease in log_2_ (fold change) ≥ 1 (treatment/control)*.

**Figure 3:**
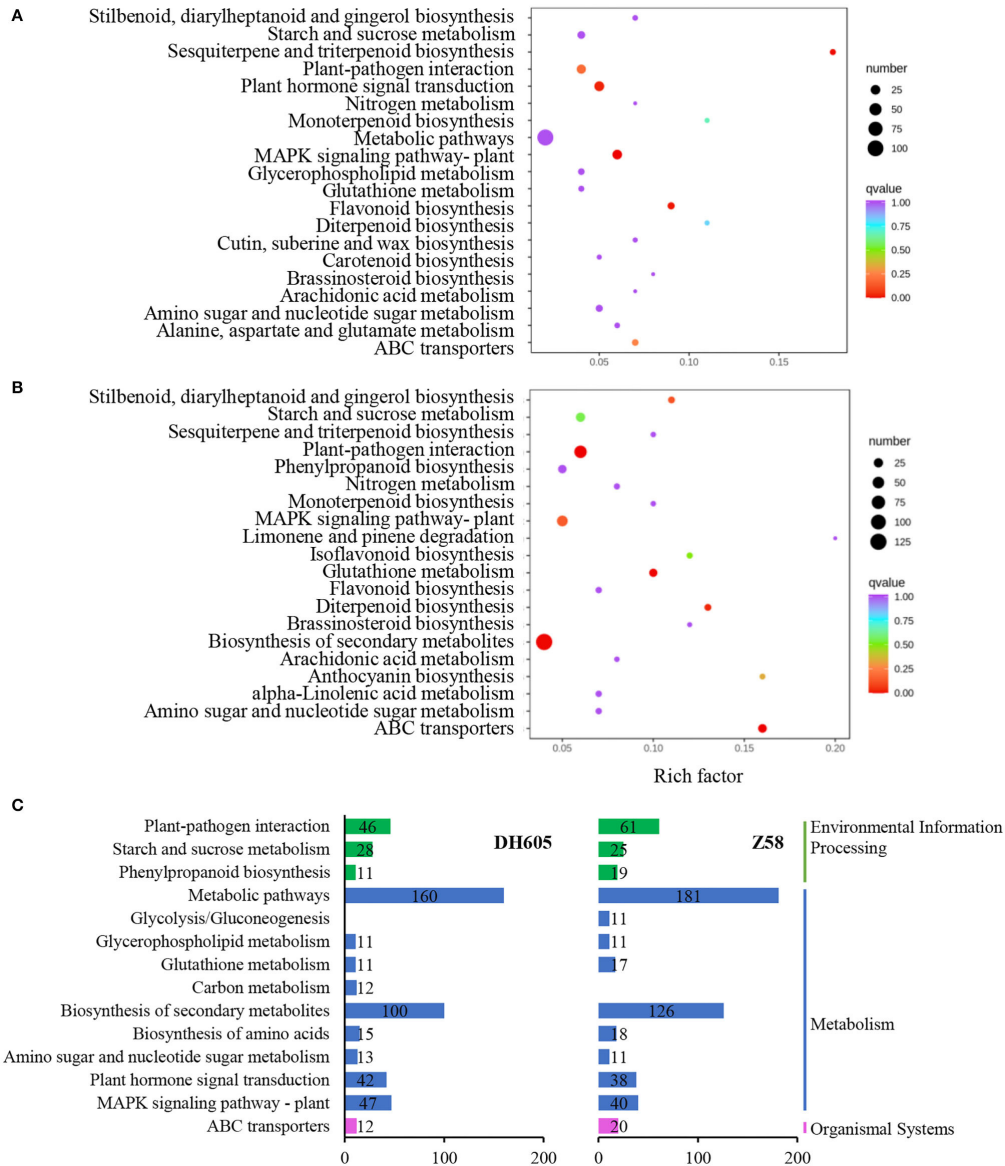
KEGG pathway analysis of DEGs under K^+^ deficiency treatment in DH605 and Z58. **(A)** KEGG pathway enrichment of DEGs under K^+^ deficiency treatment in DH605. **(B)** KEGG pathway enrichment of DEGs under K^+^ deficiency treatment in Z58. **(C)** The top-level pathways of DEGs under K^+^ deficiency treatment in DH605 and Z58 (*p* ≤ 0.05).

To gain a general understanding of the responses to K^+^ deficiency, GO and KEGG pathway enrichment analyses were performed (Figure 3, Supplementary Table 3). Among the induced pathways under the K^+^ deficiency condition, the top 15 pathways of significant enrichment are shown in Figure 3C. Most of the pathways were co-regulated in DH605 and Z58, associated primarily with regulatory processes, transport, and primary and secondary metabolism, including “ABC transporters,” “MAKP signaling pathway,” “Plant hormone signaling transduction,” “amino sugar and nucleotide sugar metabolism,” “biosynthesis of amino acids,” “starch and sucrose metabolism,” “glutathione metabolism,” and “glycerophospholipid metabolism” (Figure 3).

Transcription factors (TFs) also play pivotal roles in regulating related genes in response to stress in plants (Ulm et al., 2004). In this study, 38 transcription factors were identified for both DH605 and Z58 (Supplementary Table 4). These TFs belonged to diverse families, including AP2/ERF (2), bZIP (2), WRKY (9), MYB and MYB-related (5), NAC (3), bHLH (3), zinc finger (2), PLATZ (3), HB-HD-ZIP (2), GARP-G2-like (1), SRS (1), Tify (1), HSF (1), LOB (1), GNAT (1), and MBF1(1). Among them, the number of WRKY genes accounted for 23.6% of the total regulated TFs.

### Metabolic Responses to K^+^ Deficiency Treatment

Primary metabolites were profiled by UPLC-MS/MS to gain insight into the possible molecular metabolic mechanism of maize under K^+^ deficiency treatment, and a total of 893 kinds of metabolites were detected. Principal component analysis (PCA) was conducted on a total of 273 and 120 differential accumulated metabolites (DAMs) in DH605 and Z58, respectively (Supplementary Table 5). The PCA results showed a trend of separation among the groups (Supplementary Figure 1).

Profiled metabolites can be classified into seven categories, including amino acids, organic acids, phenolic acids, nucleotides and derivatives, sugars and sugar alcohols, alkaloids, and lipids. In general, levels of most amino acids, alkaloids, phenolic acids, and nucleotides and derivatives increased with K^+^ stress in both DH605 and Z58 (Table 2, Supplementary Table 5). The accumulated amino acids in both varieties include Ser, Val, Asn, Thr, and homoserine, as well as the non-proteinogenic amino acids such as γ-amino-butyric acid (GABA). The saccharide, like raffinose, also accumulated in shoots. Carbohydrates, such as galactinol, sucrose, maltose, and trehalose, were decreased in DH605 shoots (Supplementary Table 5a). Part of the organic acids level (L-homoserine, 4-guanidinobutyric acid, citric acid, and isocitrate) increased, while organic acids like methylene succinic acid and shikimic acid decreased in both varieties. The lipids decreased with K^+^ stress. Sugars and sugar alcohol levels, such as turanose, nicotinate D-ribonucleoside, D-glucosamine, and raffinose, increased under K^+^ stress in both varieties, whereas gluconic acid and D-saccharic acid decreased. Other sugars and sugar alcohols showed a variation under the K^+^ deficiency treatment of DH605 and Z58 shoots.

**Table 2:** Heat-map of metabolite profiles.

**Group**	**Compounds**	**DH605**	**Z58**
Amino acids and	Cycloleucine	1.02	1.16
derivatives	L-Asparagine	7.13	4.78
	L-Threonine	3.14	1.52
	L-Valine	1.37	1.41
	L-Serine	4.97	1.90
	Glutathione reduced form	2.71	3.73
	L-Ornithine	1.13	3.95
	Pipecolic acid	1.53	1.30
	L-Aspartic Acid	−3.29	−2.75
Organic acids	γ-Aminobutyric acid (GABA)	2.18	2.86
	L-Homoserine	3.16	1.33
	4-Guanidinobutyric acid	2.32	1.33
	Citric acid	2.61	1.08
	Methylenesuccinic acid	−1.48	−1.90
	Shikimic acid	−2.08	−1.36
Phenolic acids	2-Methoxy-4-ethenylphenol	1.99	1.39
	Protocatechuic acid-4-*O*-glucoside	2.57	1.13
	1-*O*-Feruloyl-3-*O*-*p*-Coumaroylglycerol	2.21	1.92
	Demethyl coniferin	1.74	1.82
	1,3-*O*-Diferuloylglycerol	3.67	1.65
	5-Hydroxymethylfurfural	5.00	2.41
	6-*O*-Caffeoyl-D-glucose	2.72	3.93
Nucleotides and	Isopentenyladenine-7-N-glucoside	1.43	1.56
derivatives	2-Deoxyribose-1-phosphate	3.62	2.90
	Xanthine	4.13	2.12
	Guanine	2.24	1.18
	Cytidine 5'-monophosphate (Cytidylic acid)	12.01	1.30
Sugars	Turanose	2.04	1.51
	Nicotinate D-ribonucleoside	2.69	2.10
	D-Glucosamine	16.78	3.35
	Raffinose	1.08	2.17
	Gluconic acid	−2.89	−3.48
	D-Saccharic acid	−1.31	−1.59
Alkaloids	N-Caffeoylputrescine	2.89	1.18
	N-*p*-Coumaroyl-N'-feruloylputrescine	4.90	3.63
	*p*-Coumaroylputrescine	1.07	1.71
	Putrescine	21.69	3.53
	Diethanolamine	14.05	2.33
	N-Acetylputrescine	2.58	3.80
	6-Deoxyfagomine	1.04	1.32
	Piperidine	1.01	1.31
	Indole	1.29	1.47
	1-Methoxy-indole-3-acetamide	1.07	1.28
Lipids	13-KODE; (9Z,11E)-13-Oxooctadeca-9,11-dienoic acid	−1.01	−1.16
	1-α-Linolenoyl-glycerol-3-O-glucoside	−1.04	−1.09
	13S-Hydroxy-9Z,11E,15Z-octadecatrienoic acid	−1.02	−2.96
	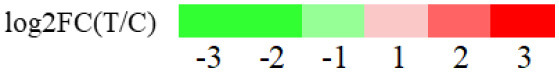		

*The value indicates metabolite changes in log2 (fold change) ≥ 1 (treatment/control). Shades of red or green indicate an increase or decrease in Log_2_FC value, respectively. Values with log_2_FC ≥1 or ≤ -1 but afflicted with a high t test are highlighted with different background colors*.

### Arginine Metabolism Under K^+^ Deficiency

**Figure 4:**
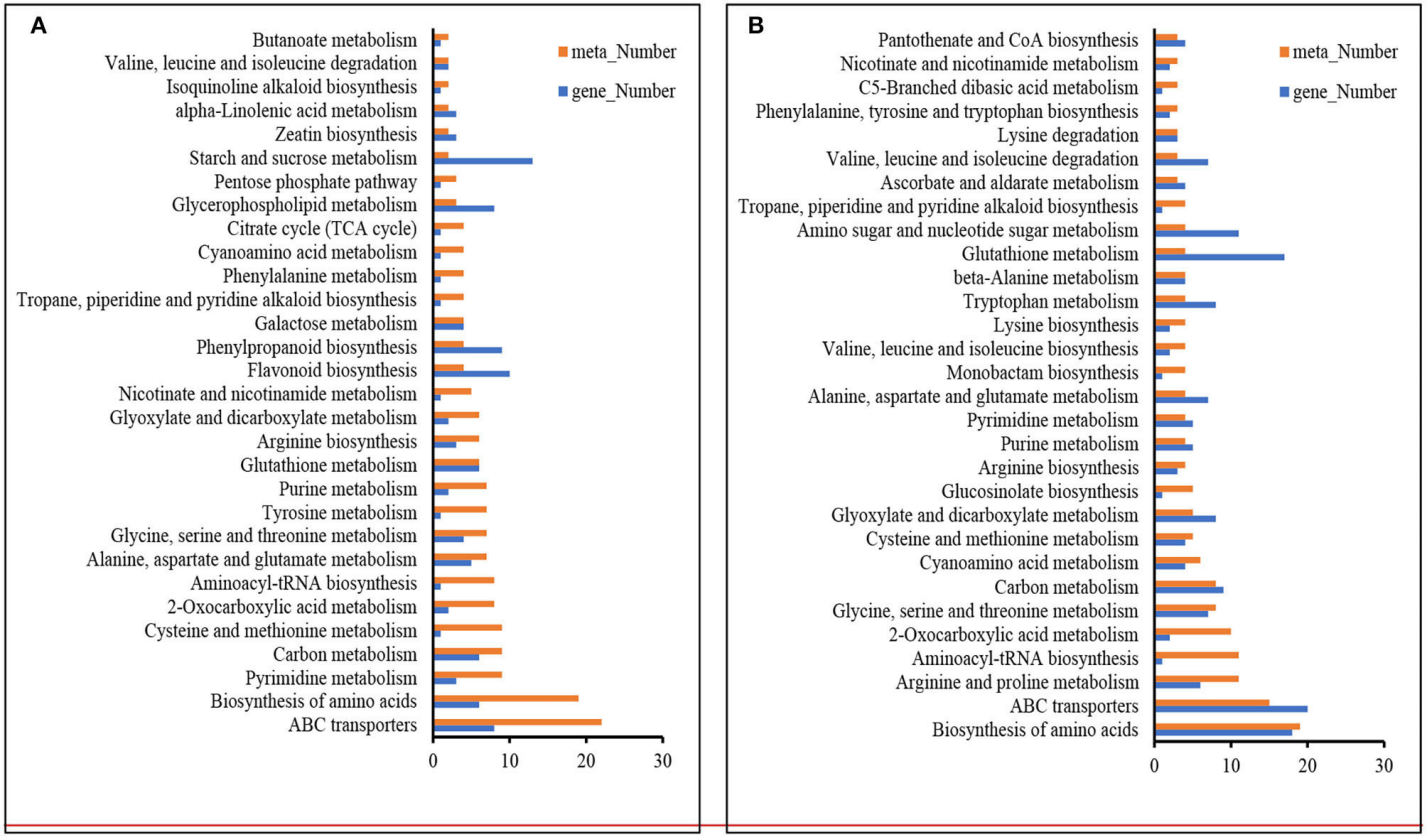
KEGG analysis of DAMs and DEGs under K^+^ deficiency treatment in DH605 **(A)** and Z58 **(B)** (*p* ≤ 0.05).

**Figure 5:**
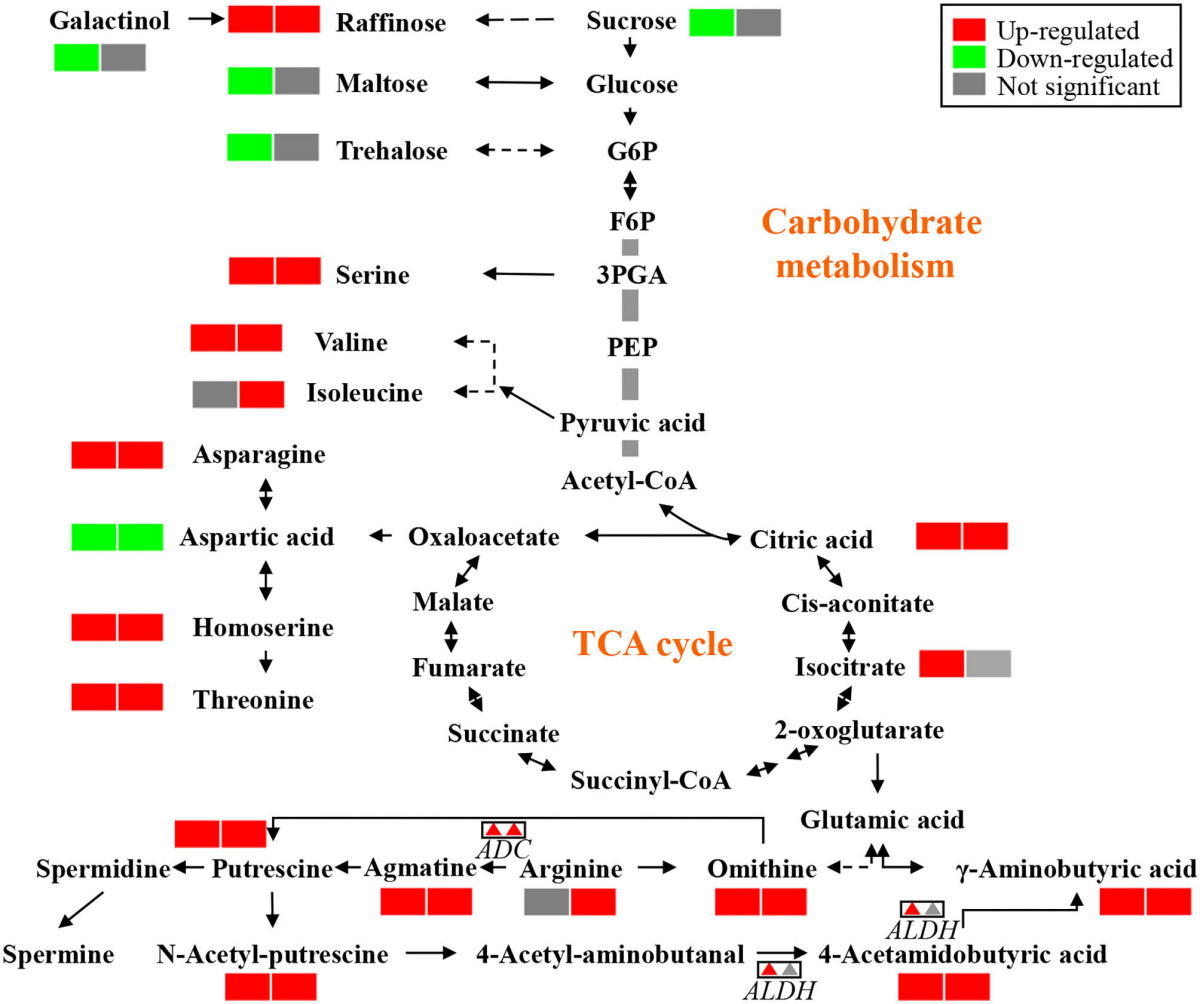
The metabolites that varied significantly in DH605 and Z58 after exposure to K^+^ deficiency. The first and second box of each metabolite indicates the varied metabolites of DH605 and Z58 after exposure to K^+^ deficiency, respectively. The first and second triangle of each gene indicates the varied genes of DH605 and Z58 after exposure to K^+^ deficiency, respectively.

Remarkably, the top 30 pathways of different accumulated metabolites included “ABC transporters,” “biosynthesis of amino acids,” “glycine, serine, and threonine metabolism,” “carbon metabolism,” “2-Oxocarboxylic acid metabolism,” and “Aminoacyl-tRNA biosynthesis” (Figure 4). Based on the above metabolome results, metabolites in arginine metabolism and the related metabolites were of interest due to their accumulation under K^+^ deficiency treatment (Figure 5). The related arginine metabolites, such as omithine, agmatine, N-Acetyl-putrescine, 4-Acetamidobutyric acid, and γ-Aminobutyric acid, were also accumulated both in DH605 and Z58 (Figure 5). Also, putrescine derivatives were accumulated as N-Caffeoyl putrescine, N-*p*-Coumaroyl-N'-feruloyl putrescine, *p*-Coumaroyl putrescine, and N-Acetyl putrescine (Table 2). Additionally, the expression of arginine decarboxylase genes (*ADC, LOC100193626*, and *LOC103638134*), mainly responsible for putrescine synthesis, were both upregulated in maize under the K^+^ deficiency condition, which was consistent with the accumulation of putrescine in maize shoots (Figure 5, Supplementary Table 2).

## Discussion

Fertilizer is important for plant growth and development and the related studies have been focused on different levels besides morphology, transcriptome, and metabolism (Pettigrew, 2008; Hafsi et al., 2014; Mo et al., 2019; Ma et al., 2020; Ding et al., 2021). In the study, the K^+^ deficiency showed a significant decrease in maize aboveground biomass production in hydroponic experiment. The transcriptome and metabolism changes under long-term K^+^ deficiency treatment were further analyzed in the hydroponic experiments. Most DEGs were classified as associated with regulatory processes, transport, and primary and secondary metabolism. DAMs induced by K^+^ deficiency were mostly involved in primary and secondary metabolism, such as amino acids and derivatives, sugars and sugar alcohols, organic acids, phenolic acids, alkaloids, and nucleotides and derivatives. The results indicated that genes related to signal transduction, including the MAKP signaling pathway and plant hormone signaling transduction, may play an important role as feedback to K^+^ limitation from the shoot to the root.

### Developmental Responses to K^+^ Deficiency

The K^+^ deficiency showed a significant decrease in silage maize grain yield and whole plant biomass and the application of K^+^ fertilizer can increase total silage maize biomass, which indicated that K^+^ directly affects maize biomass yield (Perry et al., 1972). As for maize, most studies have focused on K^+^ in maize growth and its effects on grain yield rather than plant growth (Amanullah et al., 2016; Ul-Allah et al., 2020). Due to K^+^ deficiency, plant height and stem diameter were reduced in maize, thus significantly limiting the plant growth, manifested as a decrease in fresh plant weight (Figure 1). The physiological response to K^+^ deficiency was consistent with previous studies (Kanai et al., 2007; Ma et al., 2020). K^+^ plays important role in the process of photosynthesis by converting radiant energy into chemical energy through the production of ATP, and severe K^+^ deficiency can reduce photosynthesis (Tester and Blatt, 1989; Kanai et al., 2011). Less efficient absorption of K^+^ in the root dramatically reduces its content in shoots (accounts for only 16–20% of the control), which reduces photosynthesis and inhibits plant growth (Figures 1, 2; Battie-Laclau et al., 2014; Ma et al., 2020). Moreover, the leaves and stems act as the sink of K^+^ and carbon assimilation in plant growth, and the retardation of plant growth is a feedback signal to K^+^ deficiency (Kanai et al., 2007). Similar responses to K^+^ deficiency were observed in tomato and sugarcane, as well as in wood plants *Eucalyptus grandis* (Hartt, 1969; Kanai et al., 2007; Ployet et al., 2019). Therefore, it is important to apply potassium fertilizer to improve maize biomass production in agriculture, as well as to improve the plant K^+^ utilization efficiency in breeding.

### Transcription Responses to K^+^ Deficiency

Several transcriptome profiles have been studied on plant responses to K^+^ deficiency (Ma et al., 2012, 2020; Ruan et al., 2015; Zhao et al., 2018; Ployet et al., 2019; Yang et al., 2021). The study analyzed the transcription changes of shoots in maize under K^+^ deficiency conditions, and a total of 1,638 DEGs were identified in two varieties. Many of the previously reported transcriptional K^+^ stress responses, such as K^+^ transporters and ABC transporters, were tracked in DEGs of maize shoots. K^+^ transporters play crucial roles in translocation and cell growth in various plant species (Wang and Wu, 2013). There are mainly three families in K^+^ transporters: the K^+^ uptake permeases (KT/HAK/KUP), the K^+^ transporter (Trk/HKT) family, and the cation proton antiporters (Gierth and Maser, 2007). There are 27 *HAK* genes in maize and the expression levels of *HAK1* and *HAK5* were both up-regulated under the K^+^ deficiency condition (Qin et al., 2019). The *AKT2* was also upregulated in the shoots of DH605 and Z58. The up-regulation of K^+^ transporter genes can increase K^+^ mobilization and promote the K^+^ uptake in the root as well, which would be important to redistribute and feedback the hungry status of potassium (Pilot et al., 2003; Qin et al., 2019). However, part of the *HAK* genes, like *HAK10* and *HAK11*, were downregulated in shoots. Further studies need to evaluate why the responding trend of *HAK* genes was different under K^+^ deficiency in maize shoots. Additionally, the expression of several ABC transporters was also significantly induced in shoots, indicating that ABC transporters might be important for K^+^ and its related metabolism transport in plants under low K^+^ concentrations (Xie et al., 2020). Since yellow or brown edges and tips of leaves are typical symptoms of K^+^ deficiency, DEGs involved in aging and leaf senescence were also detected (Supplementary Table 3). In addition to the above-mentioned genes, DEGs also include those involved in the biological process such as amino acid transport, amino sugar catabolic process, and response to nitrogen compounds. The shortage of K^+^ supply limits plants leaf growth, probably due to sugar starvation in sink tissues such as stem and leaves (Hafsi et al., 2014).

Transcription factors have been identified to be indispensable in biotic and abiotic stresses (Ma et al., 2012; Zhang et al., 2017). The number of differentially expressed TFs for both varieties is 38 under the K^+^ limitation condition, which included members from MYB, WRKY, bZIP, and PLATZ families (Supplementary Table 4). Part of the TFs was predicted with the MAPK signaling pathway and plant hormone signal transduction (Supplementary Table 4). The MAPK signaling pathway, which can be activated under stress conditions, was co-regulated in two varieties. It has been reported that the MAPK signaling pathway is associated with senescence and apoptosis, which could explain the leaves with yellow or brown edges and tips under K^+^ deficiency conditions (Sun et al., 2015). Besides the MAPK signaling pathway, plant hormone signal transduction was also regulated in maize under K^+^ stress. The results showed that brassinosteroid-related genes, *TCH4* (*LOC100283318* and *LOC100284736*), were downregulated in both varieties, which played important roles in cell elongation (Takahashi et al., 2005). Besides, a gene related to zeatin biosynthesis, like *type-B ARR* genes (LOC107522107 and LOC100280246), were upregulated (Argyros et al., 2008). Genes involved in plant hormone signal transduction would affect plant hormone content and distribution to regulate plant growth (Ma et al., 2020). Therefore, the MAPK signaling pathway and plant hormone signal transduction, along with the regulated TFs, may serve as critical regulators in how maize shoots respond to K^+^ deficiency.

### Metabolic Responses to K^+^ Deficiency

The responding metabolites of K^+^ stress comprised a broad range of metabolite classes, such as “ABC transporters,” “biosynthesis of amino acids,” “glycine, serine, and threonine metabolism,” “carbon metabolism,” “2-Oxocarboxylic acid metabolism,” “Aminoacyl-tRNA biosynthesis,” and so on. Of the metabolites, sugars, such as raffinose, turanose, and D-glucosamine, were accumulated, which were also detected in P deficiency and cold stress (Cook et al., 2004; Ding et al., 2021). Free amino acids (such as Asn, Thr, Val, Ser, Orn) increased in both varieties, while part of other amino acids (such as Arg, His, Leu, Pro) was only accumulated in either DH605 or Z58. K^+^ can activate enzymes and play important roles in protein synthesis (Hafsi et al., 2014). The content of amino acids increased during the K^+^ deficiency condition, while the protein content was reduced in cotton (Wang et al., 2012). Accumulation of free amino acids was also observed under P deficiency in plants (Hernandez et al., 2007; Pant et al., 2015; Mo et al., 2019; Ding et al., 2021). The results indicated that the deficiency of macronutrients in plants would suppress their carbon metabolism and affect the accumulation of amino acids. In addition, the amino acid transport-related genes responded to K^+^ deficiency, accompanied by an upregulation of genes involved in xenobiotic transmembrane transporter activity and xenobiotic transmembrane transport ATPase activity (Supplementary Table 3). However, the number of DAMs in DH605 (273) was much more than that in Z58 (120). The reason would be that DH605 are more sensitive to K^+^ deficiency and shows a severe deficiency status under 0.1 mM K^+^ treatment. Of the two varieties, which were grown on the same day in the chamber, DH605 showed a faster growing speed than Z58, with higher plant height, thicker stem diameter, and higher fresh shoot weight, also sensitive to K^+^ deficiency, the shoot K^+^ concentration.

### Comparative Transcriptome and Metabolite Responses to K^+^ Deficiency

A comparative analysis of transcriptional and metabolic responses to K^+^ deficiency showed that biosynthesis of amino acids and ABC transporters were the top two pathways regulated under K^+^ deficiency treatment. An increasing level of free amino acids indicates that plants are being damaged under low K^+^ stress, which would regulate the cellular content of K^+^ contributing to osmotolerance (Cuin and Shabala, 2007). Significantly, changes in ABC transporter genes and the accumulation of the related metabolites indicated that ABC transporters are important for K^+^ uptake and transport in maize shoots under K^+^ deficiency (Xie et al., 2020). Among the metabolites, 4-amino-butyric acid (GABA), putrescine, and putrescine derivative were significantly accumulated under K^+^ deficiency (Figure 5). Additionally, the arginine decarboxylase gene, the key gene responsible for putrescine synthesis, was upregulated under K^+^ limited conditions (Figure 5). Putrescine accumulation is one of the metabolic markers in response to K^+^ deficiency in plants, which could reduce fast-activating vacuolar (FV) channel activity under stress (Richards and Coleman, 1952; Bruggemann et al., 2002). Thus, the increased putrescine concentration and FV channel activity would maintain the cytosol K^+^ concentration (Walker et al., 1996). Putrescine is also been shown to be correlated with other stresses such as salinity and cold, as well as plant growth (Kou et al., 2018; Guo et al., 2019).

## Conclusion

This study represents a multi-level study of maize shoots' responses to K^+^ deficiency. The results suggest that the variation in transcription supports the physiological and developmental acclimation and adaptation to K^+^ deficiency. Since potassium plays important role in maize biomass yield, it is dispensable to improve the plant K^+^ utilization efficiency. The identification of differential transcriptomes and metabolomes under K^+^ deficiency and further research will be a foundation for future work in improving K^+^ utilization efficiency in the breeding program.

## Data Availability Statement

The authors acknowledge that the data presented in this study must be deposited and made publicly available in an acceptable repository, prior to publication. Frontiers cannot accept a article that does not adhere to our open data policies.

## Author Contributions

JS, XS, and WX conducted experimental design and manuscript writing. WX, YW, YP, WT, GY, YG, and JC conducted experiments and were involved in data collection. JS, XS, WX, and YW analyzed the data. All authors approved the final manuscript.

## Funding

This work was supported by grants from Forage Industrial Innovation Team, Shandong Modern Agricultural Industrial and Technical System (SDAIT-23-01), China Agriculture Research System (CARS-34), Shandong Improved Variety Program (2019LZGC002-2), and Qingdao Agricultural University high-level talent research fund (Grant No. 1120007).

## Conflict of Interest

The authors declare that the research was conducted in the absence of any commercial or financial relationships that could be construed as a potential conflict of interest.

## Publisher's Note

All claims expressed in this article are solely those of the authors and do not necessarily represent those of their affiliated organizations, or those of the publisher, the editors and the reviewers. Any product that may be evaluated in this article, or claim that may be made by its manufacturer, is not guaranteed or endorsed by the publisher.
