# Supplementary material for: Transcriptional and Metabolic Responses of Maize Shoots to Long-Term Potassium Deficiency
Source: Front Plant Sci. 2022 Jun 23;13:922581. doi: 10.3389/fpls.2022.922581 (PMC9260415; doi:10.3389/fpls.2022.922581)
Supplement: Supplementary file 1 [file Data_Sheet_1.docx]

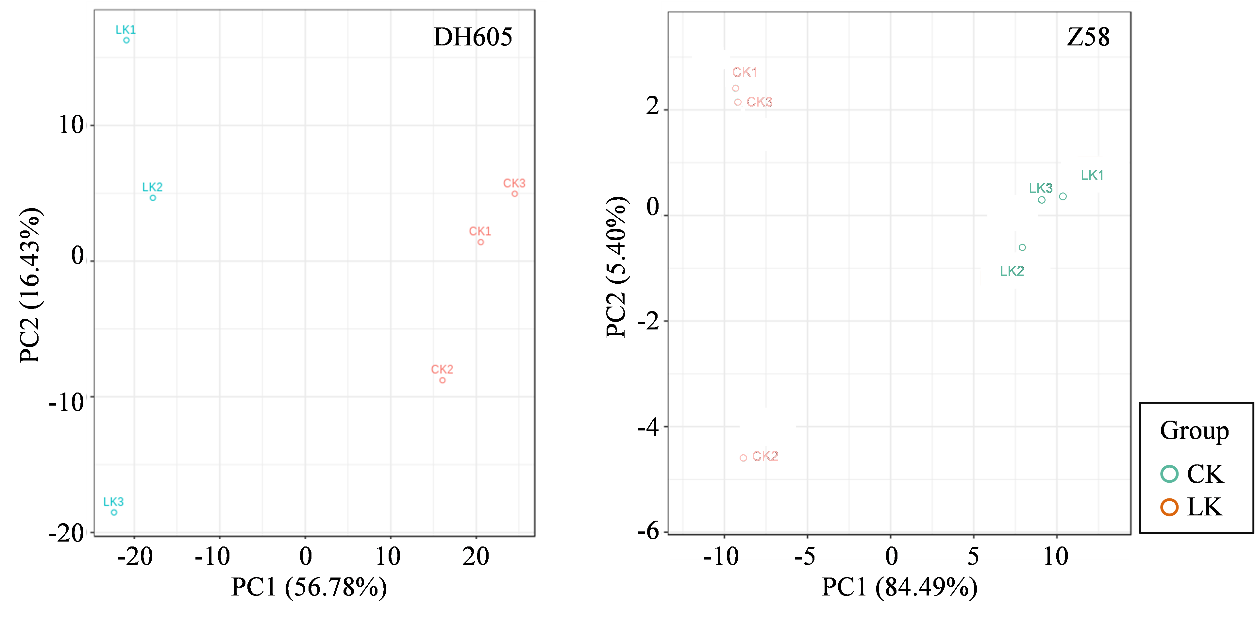


Fig. S1 Score plots of metabolic profiles in shoots of maize DH605 (left) and Z58 (right) under K^+^ deficiency by principal component analysis (PCA) method. Three biological applicates were analyzed.
